# DEPRESSION IN CAREGIVERS OF VETERANS: THE INTERACTION OF PERCEIVED SOCIAL SUPPORT AND AGE

**DOI:** 10.1093/geroni/igz038.1808

**Published:** 2019-11-08

**Authors:** Monica Scicolone, Patricia A Parmelee

**Affiliations:** The University of Alabama, Tuscaloosa, Alabama, United States

## Abstract

Caregiving is a risk factor for increased psychological stress and depression (Pinquart & Sörensen, 2003). Perceived social support (PSS) is strongly associated with emotional well-being and, for informal family caregivers, may be an important predictor of caregiver psychological outcomes. Although much is known about the effects of global PSS, there is a gap in research regarding numerous identified functional dimensions of support, particularly among family caregivers. Thus, this secondary data analysis examined how dimensions of PSS predict depression, and the moderating effects of age on this relationship, utilizing data from 240 family caregivers of elderly veterans receiving outpatient care at the Atlanta VA. The analysis utilizes a multidimensional measure of PSS (Sherbourne & Stewart, 1991) with four sub scales: emotional/informational, tangible, affectionate, and positive social interaction. Preliminary OLS regression analyses, regressing depression on relevant demographic variables and PSS domains, revealed a significant overall model (p < .001). All domains besides tangible support significantly predicted depression. Because age was the only demographic variable associated with depression, we tested hypotheses of an interaction of PSS and age with PROCESS macro (Hayes, 2017). Moderation analyses revealed a significant interaction of support and age on caregiver depression (p = .0145). Of unique social support dimensions, only emotional/informational social support interacted with age (p = .0075), demonstrating decreased depression at high levels of emotional/informational support, but a weaker effect for those of increased age. The original study was supported by the U.S. Department of Veterans Affairs Rehabilitation R&D Service.

